# Outcomes of Window Therapy with Carboplatin and Ifosfamide for Pediatric Osteosarcoma: A Case Series

**DOI:** 10.3390/children10040736

**Published:** 2023-04-17

**Authors:** Hisaki Aiba, Michi Kamei, Yasuhiko Ito, Risa Takeda, Satoshi Yamada, Hideki Okamoto, Katsuhiro Hayashi, Shinji Miwa, Yohei Kawaguchi, Shiro Saito, Takao Sakai, Hideki Murakami, Hiroaki Kimura

**Affiliations:** 1 Department of Orthopedic Surgery, Graduate School of Medical Sciences, Nagoya City University, Nagoya 467-8601, Japanhiroaki030301@gmail.com (H.K.); 2 Department of Pediatrics, Graduate School of Medical Sciences, Nagoya City University, Nagoya 467-8601, Japan; 3 Department of Pediatrics, Nagoya City University, West Medical Center, Nagoya 462-8508, Japan; 4 Department of Orthopaedic Surgery, Kanazawa University, Kanazawa 920-8641, Japan

**Keywords:** osteosarcoma, chemotherapy, surgery, carboplatin, ifosfamide

## Abstract

For the treatment of osteosarcoma, cisplatin (CDDP) can be substituted by carboplatin (CBDCA) to reduce toxicity. We report a single institution experience of CBDCA-based regimen. Two to three cycles of CBDCA + ifosfamide (IFO) therapy (window therapy) were administered as neoadjuvant therapy for osteosarcoma. Depending on the response of window therapy, the subsequent protocols were determined; for good responders, surgery is performed, and postoperative therapies with CBDCA + IFO, adriamycin (ADM) and high-dose methotrexate (MTX) were administered; for stable disease, the postoperative regimens were advanced before surgery, and the remaining amount of postoperative chemotherapy is deduced; for progressive disease, CBDCA-based regimen is changed to CDDP-based regimen. From 2009 to 2019, seven patients were treated with this protocol. During the window therapy, two patients (28.6%) were assessed as good responders and completed the regimen as planned. Four patients (57.1%) had stable disease, and the chemotherapy schedules were modified. One patient (14.2%) with progressive disease was shifted to the CDDP-based regimen. At final follow-up, four patients showed no evidence of disease and three patients died of the disease. Since the efficacy during window therapy was limited, a CBDCA-based regimen in the neoadjuvant setting was considered insufficient for performing adequate surgery.

## 1. Introduction

Osteosarcoma is the most frequently primary bone tumor in pediatric patients. Based on the data from the Surveillance, Epidemiology, and End Results Program database, the survival rates of localized osteosarcoma under 25 years old had increased from approximately 50% in the 1970s to 70% in the 1980–90s [1], and then reached a plateau of 70–80% in the 2010s [2]. During follow-up of pediatric patients, it is essential to monitor both short- and long-term toxicity associated with therapeutic agents, particularly cisplatin (CDDP), which can cause secondary malignancies, renal impairment, and ototoxicity [3,4]. To reduce the risk of cisplatin-induced nephrotoxicity, hydration, administration of diuretics or magnesium, and renal function monitoring are important [5]. Additionally, cisplatin-induced ototoxicity can lead to speech and language delays in children in a dose-dependent manner [6]. It is important to provide timely support, guidance, and access to rehabilitation services to mitigate the long-term effects of hearing loss, even if ototoxicity cannot be prevented [6].

Carboplatin (CBDCA), a second-generation platinum agent, was originally synthesized with the aim of reducing the toxicity of CDDP. Non-inferiority of CBDCA compared to CDDP has been proven in various cancers including non-small cell lung cancer and ovarian cancer, but not in bladder cancer or germ cell tumors [7]. The role of CBDCA as an alternative treatment for osteosarcoma has not yet been clarified. However, the OS-91 regimen was used at St. Jude Children’s Hospital with window therapy CBDCA and ifosfamide (IFO), and reported comparable results to those of CDDP-based chemotherapy [8,9]. In addition, IFO + CBDCA + etoposide therapy has been widely administered as a second-line treatment [10].

At our institution, we assimilated the window therapy as a modified OS-91 regimen (mOS-91) [8]. Herein, we report on the external validation of mOS-91 in a single institution.

## 2. Materials and Methods

This study was a retrospective analysis of Japanese patients treated between 2009 and 2019 at the Nagoya City University Hospital. During this period, all patients diagnosed with pediatric osteosarcoma without a previous history of chemotherapy were treated with the mOS-91 regimen. To confirm eligibility or safety for the administration of this regimen, the following criteria had to be met, both at the start of the study and initiation of every treatment session: performance status (PS) 0–2, renal function (creatine < 1.5 times the upper normal limit [UNL]), cardiac function (ejection fraction > 60%), and hepatic function (total serum bilirubin, aspartate aminotransferase, alanine aminotransferase, <1.5 times the UNL).

**Figure 1:**
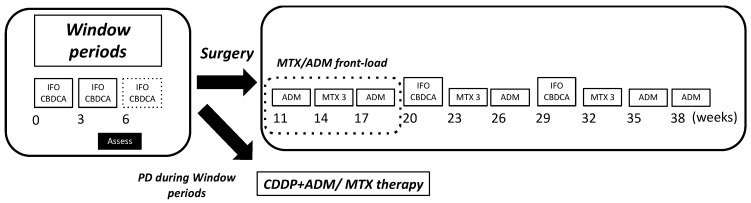
Scheme of the modified OS-91 protocol. During the window therapy, two or three cycles of CBDCA + IFO are performed before surgery. Good responders receive postoperative chemotherapy with additional CBDCA + IFO, ADM and high-dose MTX. The cumulative doses of regimens are as follows: ADM, cumulative dose = 375 mg/m^2^; high-dose MTX, cumulative dose = 108 g/m^2^; CBDCA, cumulative dose = 2.8 g/m^2^; and IFO, cumulative dose = 39.75 g/m^2^. Patients with stable disease receive an advanced postoperative regimen (ADM/MTX front-load), and after surgery, the remaining deduced amount of chemotherapy is administered. In patients with progressive disease, a CDDP-based regimen is commenced with CDDP, ADM, and high-dose MTX. ADM, Adriamycin; CBDCA, carboplatin; CDDP, cisplatin; IFO, ifosfamide; MTX, methotrexate; PD, progressive disease.

*mOS-91 regimen*: Two or three cycles of CBDCA (560 mg/m^2^ for 1 day) and IFO (2.65 g/m^2^ for 3 days, with mesna) are performed before surgery (window therapy). At 6 weeks, the response to chemotherapy is assessed using computed tomography and magnetic resonance imaging (MRI). Based on the response, the timing of surgery and postoperative chemotherapy are determined as follows. For good responders, surgery is performed after window therapy and as postoperative chemotherapy, an additional two cycles of CBDCA + IFO therapy, adriamycin (ADM) (total of 5 courses at 75 mg/m^2^ for 72-h continuous infusion), and high-dose methotrexate (MTX, total of 9 courses at 12 g/m^2^ with leucovorin rescue) are administered. For patients with stable disease, the postoperative regimens are advanced (ADM/MTX front-load; 2 ADM courses and 3 high-dose MTX courses) as preoperative chemotherapy and surgery are postponed. The remaining deduced amount of chemotherapy (3 ADM courses and 6 high-dose MTX courses) is administered postoperatively. In patients with progressive disease in window therapy, the CBDCA-based regimen is ceased and a CDDP-based regimen is commenced with an alternative CDDP therapy (100 mg/m^2^, cumulative = 400 mg/m^2^), ADM (cumulative dose = 375 mg/m^2^), and high-dose MTX (cumulative = 108 g/m^2^). Granulocyte colony-stimulating factors are appropriately used when the absolute neutrophil count is below 500/µL or 1000/µL with fever. Fluid therapy (2500–3000 mL/m^2^/day) is administered from 6 h before the start of chemotherapy until the end of chemotherapy. The protocol is shown in Figure 1. 

Response evaluation: the pathological response was evaluated using a four-tier grading system as follows: complete response with total necrosis of tumor cells (grade 4); >90% necrosis of the tumor cells (grade 3); 50–90% necrosis of the tumor cells (grade 2); and minor response with under 50% necrosis of the tumor cells (grade 1). Pathological responses were evaluated by certified pathologists in our department [11].

Preoperatively, the clinical response of the primary lesion was evaluated based on the original articles of OS-91 [8,9]. Good responders were those who were pain-free without analgesic administration and with a > 50% decrease in the sum of the products of the three perpendicular tumor diameters. Patients with progressive disease had a > 25% increase in the sum of the products of the three perpendicular tumor diameters or new lesions. The remaining patients were considered to have stable diseases.

Toxicity: the toxicity of this protocol was evaluated based on the Common Terminology Criteria for Adverse Events version 5. Short-term toxicity was counted separately during each session. Pure-tone audiometry was evaluated before induction of chemotherapy and at the end of chemotherapy, followed by annual routine check-ups. Leukoencephalopathy was evaluated using MRI at the end of chemotherapy. For short- or long-term toxicity for kidney function, creatinine clearance via 24-hr urine collection and the levels of urine protein, albumin, N-Acetyl-β-d-Glucosaminidase and serum cystatin C, creatinine were evaluated [12]. In addition, cardiac function was routinely evaluated via echocardiography, and assessment of serum levels of brain natriuretic peptide. 

## 3. Results

During this period, seven patients received the mOS-91 protocol. The median age of the patients at initial diagnosis was 12.9 years (range, 8.3–14.5). Six patients were male and one was female. The locations of the tumors were as follows: the distal femur in four patients, proximal humerus in one patient, distal tibia in one patient, and pelvis in one patient. In terms of subtype, five patients showed an osteoblastic subtype, one patient showed a chondroblastic subtype, and one patient showed a telangiectatic subtype. The American Joint Committee on Cancer/International Union Against Cancer (eighth edition) stage was stage IIa in one patient, stage IIb in two patients, stage III in three patients, and stage IVb in one patient. The median follow-up period was 28 months (range, 16–166 months). The Table S1 provides the detailed information.

**Figure 2:**
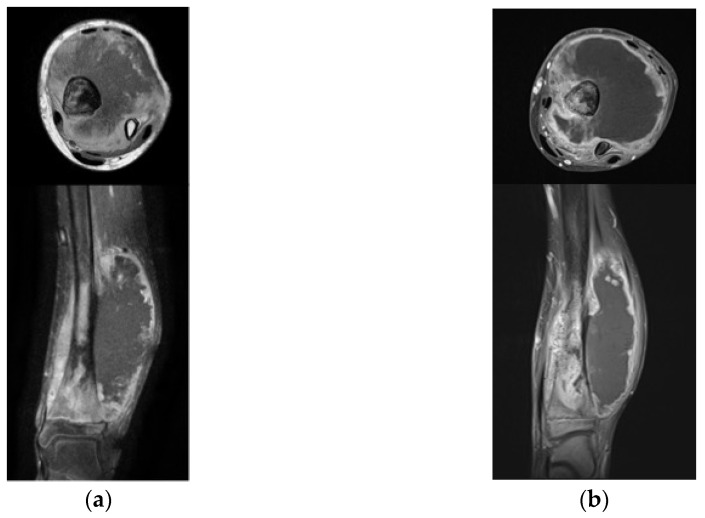
Representative case 1. The patient (patient No. 6) presented with a lytic lesion and peritoneal reaction at the distal tibia (**a**). Biopsy revealed conventional osteosarcoma, and the patient underwent CBDCA + IFO chemotherapy. After the window therapy, the tumor shrunk (assessed as good responder); however, due to the progression around the nerve-vessel bundles, amputation below the knee joint was performed (**b**). After surgery, the patient completed the mOS-91 regimen, and there is currently no evidence of disease. CBDCA, carboplatin; IFO, ifosfamide; mOS-91, modified OS-91.

Regarding response during the window therapy, two patients (28.6%) were assessed as good responders and completed the regimen as planned (Figure 2, patient No. 6).

**Figure 3:**
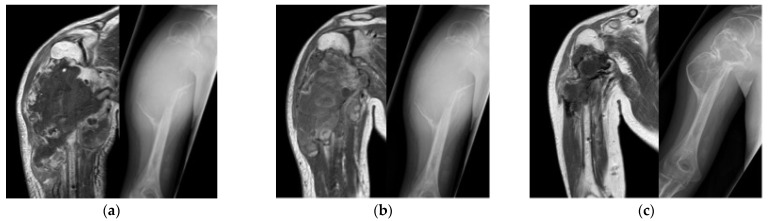
Representative case 2. The patient (patient No. 2) was referred to our hospital due to swelling of right shoulder. Based on the biopsy results, the patient was diagnosed with conventional osteosarcoma. The radiography at the initial visit (**a**). After induction of window therapy with CBDCA + IFO, the size of the tumor did not change (**b**). Thus, ADM/MTX was administered before surgery (ADM/MTX front-load). After several additional cycles of chemotherapy, the tumor responded to chemotherapy (**c**); however, an amputation was recommended due to the invasion of the neurovascular bundles. After surgery, the patient completed the mOS-91 regimen, and there is currently no evidence of disease. ADM, Adriamycin; CBDCA, carboplatin; IFO, ifosfamide; mOS-91, modified OS-91; MTX, methotrexate.

Four patients (57.1%) were assessed as having a stable disease during this period based on images; thus, they underwent ADM/MTX front-load therapy (Figure 3, patient No. 2). Due to the progression of the primary lesion, one patient (14.2%) was changed to the CDDP-based regimen. 

Limbs were not able to be preserved in five patients (71.4%). Of the resected specimens, two patients who completed the regimen on schedule showed a grade 3 response. In the ADM/MTX front-load group, one patient showed a grade 1 response, two patients showed a grade 2 response, and one patient showed a grade 3 response. The patient who was shifted to the CDDP regimen showed a grade 3 response. 

In terms of the oncological outcomes at final follow-up, four patients showed no evidence of disease and three patients died of the disease. Local recurrence occurred in one patient (patient No. 1) who subsequently underwent proton beam therapy. 

The toxicity during the window therapy is shown in Table 1. Although severe hematologic toxicity occurred frequently, it was resolved with granulocyte colony-stimulating factor and blood transfusion. During this period, severe cardiac disorders, kidney disorders, and hepatobiliary disorders did not occur. Regarding mid-term toxicity, the mean cardiac function (ejection fraction) changed from 65.4 ± 5.7% (at the initiation of treatment) to 62.2% ± 7.0 (at the end of treatment). 

## 4. Discussion

CBDCA is a synthesized-platinum compound containing bidentate dicarboxylate. Compared with CDDP, CBDCA allegedly has fewer side effects, especially in renal disorders [13] and hearing loss [14]. As the life-threatening late-onset side effects due to anti-tumor chemotherapy are not negligible, clinicians should manage these problems from a long-term perspective.

Originally, the OS-91 regimen was administered to 69 newly diagnosed patients with osteosarcoma. The clinical and radiographic response rate in the window therapy was 67.7% and the grade 3–4 histological response rate was 56.3% [8]. Among the patients with localized lesions, the 3-year survival rate was 76.4% [8]. In this study, we used a modified version of the OS-91 regimen. As a merit of this version, based on responses during the window therapy, the change of the protocol to ADM/MTX front-load or CDDP treatment was properly permitted. We encountered several patients who responded poorly to window therapy but were eventually managed by a shift to ADM/MTX front-load and good response (Figure 3). As it is widely reported that the histological response is one of the significant factors contributing to oncological outcomes [15], the modifications of the regimen with expectations of an improved response to chemotherapy were considered important.

EURAMOS-1 trial analyzed 2260 patients who were diagnosed with resectable osteosarcoma, and poor responders (>10% viable tumor in resected specimens) were randomly assigned to a MAP (MTX + ADM + CDDP) or MAP/IFO + etoposide regimen [16]. This trial revealed that the histological response to induction chemotherapy was an important factor in relapse. Switching to IFO-based chemotherapy, however, did not improve the outcomes, and increased toxicity [16]. Similarly, in Japan, the neoadjuvant chemotherapy for osteosarcoma (NECO) study—a multi-institutional prospective phase II study—was conducted from 1993 to 2001 [17]. This protocol was initiated by MAP therapy, followed by CDDP and ADM. After one cycle of MAP, patients assessed as having a progressive disease were converted to a high-dose IFO-based regimen. The rate of progressive disease after one cycle of MAP was approximately 18%, and histologically good responders accounted for approximately 42% of the group of patients. The 5-year survival rate was 77.9%, and there was no significant difference between histologically good responders and poor responders, probably due to the salvage of poor responders with an IFO-based regimen [17]. Currently, a new study (JCOG0905 trial) is underway to evaluate MAP regimens with and without the addition of IFO for the Japanese population [18].

Similar to the current protocol, a study conducted in Thailand administered CBDCA + DOX-based neoadjuvant chemotherapy with eight cycles (four preoperative, four postoperative) of CBDCA (400 mg/m^2^) + DOX (60 mg/m^2^) [19]. Unexpectedly, inadequate histological responses (less than 90% necrosis) occurred in the majority of patients. Three-year survival rate and disease-free survival rate were 47.1% and 20.2%, respectively. The authors concluded that the oncologic outcomes were inferior to the widely accepted regimen due to the insufficient effects of CBDCA and that refinement of the protocol should be considered in future protocols [19].

As for side effects, kidney dysfunction is sometimes caused by CDDP or IFO-associated glomerular dysfunction or tubular toxicity [20]. The sequelae require long-term electrolyte supplementation to prevent irreversible renal failure [21]; an IFO dose of 45 g/m^2^ is considered to be a risk factor for kidney insufficiency. The previous protocols performed with MAP treatments in the EURAMOS-1 trial revealed that the short-term renal toxicities (grade 1–2) were 14%–18% and 1–2% (grade 3–4) [16]. On the other hand, in the OS-91 protocol, 5% of patients developed renal toxicity (grade 3–4), but no patients had permanent sequelae [8]. A similar tendency was observed for the current regimen.

In addition, hearing loss is caused by irreversible damage to the hearing cells due to CDDP or less frequently due to CBDCA (equivalent to one-quarter CDDP) [22]. Averting high peak CDDP concentration via the arrangement of the schedule is important for reducing the risk of inner ear damage [22]. This problem might interrupt patients’ understanding due to the deterioration of speaking or listening skills. To minimize hearing deterioration and disability, routine check-ups of hearing skills are important [23]. In the current study, we did not observe any deterioration in hearing loss. This tendency was identical to that of the original OS-91 regimen [8].

There were some limitations associated with the protocol used in this study. First, some patients did not respond to chemotherapy or exhibited tumor progression during the window therapy. Compared with the original OS-91 regimen, the response to chemotherapy during the window therapy was inferior in the current series (28.5% [current] vs. 67.7% [original]). This might be partially due to the difficulty in precise evaluation of the effectiveness of preoperative chemotherapy in osteosarcoma. Second, we administered 7.95 g/m^2^ of IFO per protocol and 39.75 g/m^2^ as a total dose. This accumulation is below that reported by the EURAMOS-1 study (total 60 g/m^2^ for poor responders) [21] and the JCOG0905 study (15 g/m^2^ per protocol, total 90 g/m^2^ for poor responders) [18]. In addition, the dose of CBDCA was not adjusted according to kidney function or the area under the curve, which is in accordance with the original OS-91 regimen, resulting in a lack of adjustment of the accurate dose of CBDCA. This may have led to insufficient efficacy during CBDCA + IFO window therapy.

## 5. Conclusions

We performed preoperative chemotherapy with window therapy of CBDCA + IFO therapy. Due to the insufficient efficacy during the window therapy, treatment strategies were changed to alternative schedules or regimens in some cases. Chemotherapy efficacy in the neoadjuvant setting is important for performing adequate surgery aimed at limb-sparing; thus, chemotherapy with window therapy of CBDCA + IFO therapy may be less useful.

## Figures and Tables

**Table 1:** Toxicity during window therapy with carboplatin and ifosfamide.

Grade 3/4 Toxicity (15 Cycles in 7 Patients)		The Number of Events
Blood and lymphatic disorders	Neutropenia	13
	Anemia	11
	Thrombocytopenia	8
	Febrile neutropenia	2
Renal and urinary/metabolism disorders	Creatinine elevation	0
	Electrolyte imbalance	0
Gastrointestinal disorders/hepatobiliary disorders	Vomiting	0
	Nausea	0
	Anorexia	0
	Alanine/aspartate aminotransferase increased	0
	Increased blood bilirubin level	0
Cardiac disorders	Heart failure	0

## Data Availability

We provided details of data in the supplemental table.

## Supplementary Materials

The following are available online at https://www.mdpi.com/article/10.3390/children10040736/s1, Table S1: Patients’ characteristics.
