# Supplementary material for: Outcomes of Window Therapy with Carboplatin and Ifosfamide for Pediatric Osteosarcoma: A Case Series
Source: Children (Basel). 2023 Apr 17;10(4):736. doi: 10.3390/children10040736 (PMC10136696; doi:10.3390/children10040736)
Supplement: Supplementary file 1 [file children-10-00736-s001.zip › children-2269621-supplementary.pdf]

Supplemental Table S1. Patients' characteristics

Number	Location	Age (years)	Sex	Subtype	Response during window period	Histological response	Treatment	Surgery	Outcome	Details
1	Pelvis	12.9	Female	Chondroblastic	SD	Grade 4	On schedule	Wide resection	DOD	Recurrence at 10 months after mOS-91. As second-line treatment, ICE was administered and proton beam irradiation was performed.
2	Proximal humerus	14.6	Male	Osteoblastic	SD	Grade 2	MTX/ADM front-load	Amputation	NED	Responded to MTX/ADM front-load. Presented in Figure 3.
3	Distal femur	8.3	Male	Osteoblastic	SD	Grade 2	MTX/ADM front-load	Amputation	NED	Remained stable after alternation to MTX/ADM front-load.
4	Distal femur	13.0	Male	Osteoblastic	PD	Grade 3	MTX/ADM front-load	Amputation	DOD	In the window therapy, due to the progression of the disease, spinal metastasis was pointed out.
5	Distal femur	8.5	Male	Osteoblastic	PD	Grade 1	MTX/ADM front-load	Amputation	DOD	Progressed after alternation to MTX/ADM front-load.

6	Distal tibia	12.9	Male	Osteoblastic	PR	Grade 3	On schedule	Amputation	NED	Presented in Figure 2.
7	Distal femur	13.8	Male	Telangiectatic	PD	Grade 3	Change to CDDP-based regimen	Wide resection	NED	Responded to CDDP-based regimen

MTX, methotrexate; ADM, adriamycin; CDDP, cisplatin; SD, stable disease; PR, partial response; PD, progressive disease; DOD, died of disease; NED, no evidence of disease; mOS-91, modified mOS-91 regimen; IFO, ifosfamide; CBDCA, carboplatin; ICE, IFO + CBDCA + etoposide therapy
